# Clinical Usefulness of Anthropometric Indices to Predict the Presence of Prediabetes. Data from the ILERVAS Cohort

**DOI:** 10.3390/nu13031002

**Published:** 2021-03-19

**Authors:** Marta Sánchez, Enric Sánchez, Marcelino Bermúdez-López, Gerard Torres, Cristina Farràs-Sallés, Reinald Pamplona, Eva Castro-Boqué, José Manuel Valdivielso, Francisco Purroy, Montserrat Martínez-Alonso, Pere Godoy, Dídac Mauricio, Elvira Fernández, Marta Hernández, Ferran Rius, Albert Lecube, On Behalf of the ILERVAS Project Collaborators

**Affiliations:** 1 Endocrinology and Nutrition Department, University Hospital Arnau de Vilanova, Obesity, Diabetes and Metabolism (ODIM) Research Group, IRBLleida, University of Lleida, Rovira Roure 80, 25198 Lleida, Spain; ma.san.pe.88@gmail.com (M.S.); esanchez@irblleida.cat (E.S.); martahernandezg@gmail.com (M.H.); friusriu@gmail.com (F.R.); 2 Vascular and Renal Translational Research Group, IRBLleida, RedinRen-ISCIII, University of Lleida, 25198 Lleida, Spain; mbermudez@irblleida.cat (M.B.-L.); ecastro@irblleida.cat (E.C.-B.); valdivielso@irblleida.cat (J.M.V.); efernandez@irblleida.cat (E.F.); 3 Respiratory Department, University Hospital Arnau de Vilanova-Santa María, Translational Research in Respiratory Medicine, IRBLleida, University of Lleida, 25198 Lleida, Spain; gtorres@gss.scs.es; 4 Centro de Investigación Biomédica en Red de Enfermedades Respiratorias (CIBERES), Instituto de Salud Carlos III (ISCIII), 28029 Madrid, Spain; 5 Applied Epidemiology Research Group, IRBLleida, 25007 Lleida, Spain; cfarras.lleida.ics@gencat.cat (C.F.-S.); pere.godoy@gencat.cat (P.G.); 6 Institut Català de la Salut, Unitat de Suport a la Recerca Lleida, Institut Universitari d’Investigació en Atenció Primària Jordi Gol (IDIAP Jordi Gol), 25007 Lleida, Spain; 7 Experimental Medicine Department, IRBLleida, University of Lleida, 25198 Lleida, Spain; reinald.pamplona@mex.udl.cat; 8 Stroke Unit, University Hospital Arnau de Vilanova, Clinical Neurosciences Group, IRBLleida, University of Lleida, 25198 Lleida, Spain; fpurroygarcia@gmail.com; 9 Systems Biology and Statistical Methods for Biomedical Research Group, Biostatistics Unit, IRBLleida, Universitat de Lleida, 25198 Lleida, Spain; mmartinez@irblleida.cat; 10 Department of Endocrinology and Nutrition, Hospital de la Sant Creu i Sant Pau, Sant Quintí, 08041 Barcelona, Spain; didacmauricio@gmail.com; 11 Centro de Investigación Biomédica en Red de Diabetes y Enfermedades Metabólicas Asociadas (CIBERDEM), Instituto de Salud Carlos III (ISCIII), 28029 Madrid, Spain

**Keywords:** adiposity, body composition, prediabetes, glycated hemoglobin, obesity

## Abstract

Prediabetes is closely related to excess body weight and adipose distribution. For this reason, we aimed to assess and compare the diagnostic usefulness of ten anthropometric adiposity indices to predict prediabetes. Cross-sectional study with 8188 overweight subjects free of type 2 diabetes from the ILERVAS project (NCT03228459). Prediabetes was diagnosed by levels of glycated hemoglobin (HbA1c). Total body adiposity indices [BMI, Clínica Universidad de Navarra-Body Adiposity Estimator (CUN-BAE) and Deurenberg’s formula] and abdominal adiposity (waist and neck circumferences, conicity index, waist to height ratio, Bonora’s equation, A body shape index, and body roundness index) were calculated. The area under the receiver-operating characteristic (ROC) curve, the best cutoff and the prevalence of prediabetes around this value were calculated for every anthropometric index. All anthropometric indices other than the A body adiposity were higher in men and women with prediabetes compared with controls (*p* < 0.001 for all). In addition, a slightly positive correlation was found between indices and HbA1c in both sexes (r ≤ 0.182 and *p* ≤ 0.026 for all). None of the measures achieved acceptable levels of discrimination in ROC analysis (area under the ROC ≤ 0.63 for all). Assessing BMI, the prevalence of prediabetes among men increased from 20.4% to 36.2% around the cutoff of 28.2 kg/m^2^, with similar data among women (from 29.3 to 44.8% with a cutoff of 28.6 kg/m^2^). No lonely obesity index appears to be the perfect biomarker to use in clinical practice to detect individuals with prediabetes.

## 1. Introduction

The occurrence of prediabetes has expanded progressively in recent decades, reaching the 374 million people affected in 2017 according to the International Diabetes Federation [1]. It is clear that subjects with this metabolic condition are estimated to have a 40% to 50% risk of developing type 2 diabetes [2]. In addition, prediabetes is also associated with an accumulation of metabolic abnormalities, an increased risk of microangiopathy and cardiovascular disease, dementia, cancer, and lung dysfunction that occur before fasting plasma glucose reaches the threshold diagnosis of type 2 diabetes mellitus [3,4,5,6]. Therefore, So, this long asymptomatic stage needs more attention, and the detection of prediabetes in patients with a high metabolic risk should be considered [7]. One way to test this screening may be by assessing body composition.

Prediabetes is closely related to excess body weight and adipose distribution in several cross-sectional and longitudinal studies [8,9,10,11,12,13]. The China National Diabetes and Metabolic Disorder Study, which recruited 10,098 men and 17,545 women, observed how body mass index (BMI) and waist circumference (WC) were clearly associated with the prevalence of prediabetes [11]. Similarly, a strong association between WC and prediabetes was also detected in 2022 Spanish subjects [14]. However, BMI and WC categories are far from perfect for providing an accurate assessment of the amount and distribution of [15,16]. Because reference techniques for directly measuring total adipose tissue and abdominal adiposity are complex, costly and time-consuming, their widespread use in clinical practice is limited [17]. Therefore, to replace dual-energy x-ray absorptiometry (DXA) and magnetic resonance imaging, more than a few mathematical indices that combine anthropometric data have been proposed to assess total body fat and abdominal adiposity [18,19,20,21,22,23,24]. Data on the predictive capacity of prediabetes of some of these indices are scarce or absent.

On this basis, the main objective of this study was to determine and compare the diagnostic performance of adiposity estimated by different indices in the prediabetes stage in a large cohort of middle-aged overweight participants with low to moderate cardiovascular risk. For this purpose, we calculated ten anthropometric indices (three related with total adiposity and seven of central adiposity). The study was done stratified by sex, as its disproportion in body fatness and adiposity distribution has been well documented [25].

## 2. Materials and Methods

### 2.1. Design of the Study and Description of the Study Population

The ILERVAS project (ClinTrials.gov (accessed on 21 November 2020) Identifier: NCT03228459) comprises a complete cohort of 8330 participants enrolled between January 2015 and December 2018 from 32 primary health care centers from the Catalan Health Institute in the province of Lleida, Spain [26,27]. The ILERVAS project is a prospective study dealing with the benefits of the early diagnosis of subclinical atheromatous disease and undiagnosed kidney disease in a population with low to moderate cardiovascular risk. Inclusion criteria for eligible patients were between 45 and 70 years of, with no previous cardiovascular disease, with at least one cardiovascular risk factor [dyslipidemia, hypertension, obesity, smoking habit or a first-degree relative with premature (<55 years old in men, <65 in women) cardiovascular disease (myocardial infarction, stroke and peripheral arterial disease)]. The exclusion criteria were any type of diabetes, kidney disease, active neoplasia, life expectancy of less than eighteen months and/or pregnancy.

### 2.2. Prediabetes Screening

According to *American Diabetes Association guidelines*, normal glucose metabolism was well-defined as HbA1c < 39 mmol/mol (<5.7%), and prediabetes as HbA1c between 39 and 47 mmol/mol (5.7 to 6.4%) [7]. The evaluation of HbA1c was done in capillary blood using a point-of-care device (Cobas B 101^®^, Roche Diagnostics S.L., Sant Cugat del Vallès, Spain), based on a latex agglutination inhibition immunoassay method that meets the generally recognized performance criteria for HbA1c [28]. One hundred and forty-two individuals with type 2 diabetes previously undiagnosed (HbA1c ≥48.0 mmol/mol (≥6.5%)) were excluded from the research that was finally performed in 8188 subjects.

### 2.3. Anthropometry and Calculation of Indices

Weight and height were determined in light clothing and without shoes using standard tools, to the closest 0.5 kg and 1.0 cm, respectively. BMI was defined as body weight (kg) divided by the square of the body height (m), and obesity was defined as a BMI ≥30 kg/m^2^ [29]. A non-elastic tape with an accuracy of 0.1 cm was used to assess the circumferences of the waist and neck. WC was evaluated with the subject in a standing position, in the horizontal plane between the iliac crest and the lowest rib [30]. Neck circumference was assessed in the midway of the neck in a plane as horizontal as possible, with subjects standing upright. In men with a laryngeal prominence, it was assessed just below prominence [31]. All anthropometrical measurements were made by trained nurses under standardized conditions to avoid inter-observer and inter-device variability.

Two indices were added to BMI to evaluate total body fat. The Clínica Universidad de Navarra—Body Adiposity Estimator (CUN-BAE) was calculated as follow: −44.988 + (0.503 × age) + (10.689 × sex) + (3.172 × BMI) − (0.026 × BMI^2^) + (0.181 × BMI × sex) − (0.02 × BMI × age) − (0.005 × BMI^2^ × sex) + (0.00021 × BMI^2^ × age), where sex is 1 for females and 0 for males, and age in years [18]. The formula recommended by Deurenberg et al. considers body fat using the equation: (1.20 × BMI) + (0.23 × age) − (10.8 × sex) − 5.4, where female is 0 and male is 1 for sex [19].

Along with the waist and neck circumferences, five equations for central adiposity were included as a test: (i) the conicity index suggested by Valdez et al. in 1991, (ii) the waist to height ratio (WHR), (iii) the equation proposed by Bonora in 1995, (iv) the A body adiposity index established by Krakauer et al. in 2012, and (v) the body roundness index developed in 2013 by Thomas et al. [20,21,22,23,24]. The conicity index is created on the hypothesis that persons that accumulate abdominal fat have a silhouette like a double cone (that is, two cones sharing the same base, one positioned over the other), whereas people with less visceral adiposity have the shape of a cylinder. Therefore, conicity index ranges from 1.0 (a perfect cylinder) to 1.73 (a perfect double cone) and is attained according to the next formula: 0.109^−1^ × WC (m) × [weight (kg)/height (m)]^−1/2^ [20].

The WHR was measured as WC (m) divided by height (m) [21]. The equation sugested by Bonora et al. uses diverse formulas depending on sex: −453.7 + (6.37 × WC) for men, and −370.5 + (4.04 × WC) + (2.62 × age) for women [22]. To assess the A body shape index the eccentricity of the body (ε) needs to be determined [23]. The ε is a non-dimensional number that computes the degree of circularity of an ellipse, ranges from zero (perfect circle) to one (a vertical line) and is evaluated by the formula: [1 − π^−2^ × WC (m)^2^ × height (m)^−2^]^1/2^. After, the following formula measures the A body shape index: 364.2 − (365.5 × ε), in which values closer to 1 are related to rounder individuals, whereas lower values are associated with leaner individuals. Finally, the body roundness index was calculated as: WC (m)/[BMI^2/3^ × height (m)^1/2^] [24].

As hip circumference was not included in the initial design of the ILERVAS study, anthropometric indices containing hip circumference such as abdominal volume index, body adiposity index, and waist to hip ratio were not reflected in our study. The short version of The International Physical Activity Questionnaire (IPAQ) was administered to all participants. The metabolic equivalent of task (METs)-min per week, a multiple of the resting metabolic rate, was assessed [32].

### 2.4. Statistical Analysis

Participants were classified based to the presence of prediabetes. The normal distribution of the variables was evaluated using the Shapiro-Wilk test. Known for its skewed distribution, quantitative data were showed as the median [interquartile range]. Baseline characteristics through the diagnosis of prediabetes were analyzed using the Mann-Whitney U test for quantitative variables, and the Pearson’s chi-squared for categorical variables. The risk ratio was shown. The bivariate interactions between obesity indices were evaluated by the Spearman correlation test. Because differences in the amount and distribution between men and women are recognized, we showed our results by sex [31].

The areas under Receiver Operating Characteristic (ROC) curves with a whole sensitivity/specificity report and calculating Youden J statistic were designed to study the diagnostic performance of all anthropometric indices to discriminate prediabetes. In addition, a new variable obtained from the combination of anthropometric indices (BMI, Deuremberg, WHR, conicity index, body roundness index, and neck circumference) significantly related with the presence of prediabetes in the logistic regression model (backward method) was calculated. An odds ratio with its 95% confidence interval was also calculated. The area under the ROC curve was interpreted following the next guidelines: 0.9 to 1.0, excellent; 0.8 to 0.9, good; 0.7 to 0.8, fair; 0.6 to 0.7, poor; and 0.5 to 0.6, not useful. All statistical analyses were made via the SSPS statistical package (IBM SPSS Statistics for Windows, Version 20.0. Armonk, NY, USA). All *p*-values were based on a two-sided test of statistical significance, set at *p* < 0.050.

### 2.5. Ethics Statement

Informed written consent was obtained from all subjects, and the ILERVAS protocol was approved by the ethics committee of the Arnau de Vilanova University Hospital (CEIC-1410). The investigation was completed agreeing with the ethical guidelines of the Declaration of Helsinki and also followed Spanish legislation concerning the protection of personal data.

## 3. Results

The ILERVAS cohort comprises 2731 (33.4%) individuals with prediabetes. The main clinical and metabolic data according to the presence of prediabetes are displayed in Table 1. Individuals with prediabetes were mainly women with a characteristic adverse cardiovascular risk profile, including a higher age and a higher prevalence of blood hypertension and dyslipidemia than participants with a normal glucose metabolism. In addition, participants with prediabetes also showed an increased prevalence of obesity (40.1 vs. 29.0%, *p* < 0.001).

In both sexes, as well as in the entire population, prediabetes was associated with significantly higher values of total and visceral adiposity indices, with the exception of the A body shape index (Table 2 and Supplementary Table S1). Similarly, HbA1c showed a slightly but significant positive correlation with all anthropometric indices apart for the A body shape index among participants with prediabetes (Table 3). No differences were observed in total MET-minutes/week between the groups (495 (0–1200) vs. 495 (0–1200), *p* = 0.984).

**Figure 1:**
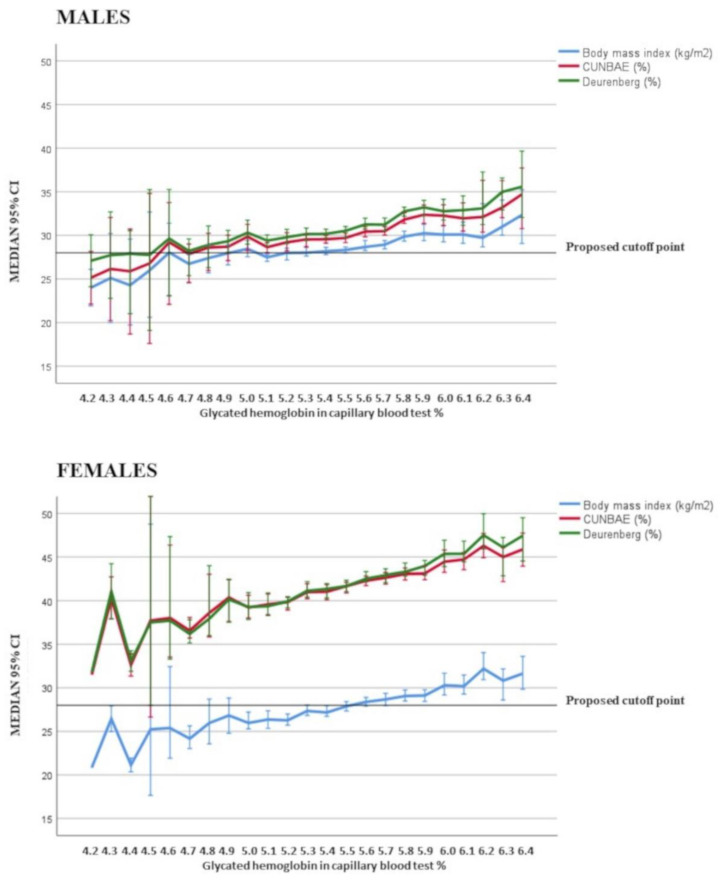
Results of the total adiposity indices in male and female individuals according to the level of glycated hemoglobin value.

ROC analysis showed that, in our population, measures related to total body fat and abdominal adiposity had a significant but fair power to identify patients with prediabetes (Table 4 and Supplementary Table S2). The best cutoff for each index, which combines sensitivity plus specificity, is available in Table 4. Taking BMI as an example, the prevalence of prediabetes among men increased from 20.4% to 36.2% around the cutoff of 28.2 kg/m^2^ (odds ratio 2.2 [95% CI 1.9 to 2.5]; <0.001), with similar data among women (from 29.3 to 44.8%; OR 2.0 [95% CI 1.7 to 2.2]; *p* < 0.001) (Figure 1). Similar data for the remaining anthropometric indices are shown in Table 5. The area under the ROC obtained with the new variable calculated by including six of the selected anthropometric indices was 0.63 [95% CI 0.62 to 0.64], similar to that of separate indices.

## 4. Discussion

In the middle-aged Caucasian ILERVAS cohort, participants with prediabetes showed a significant increase in estimated total body and abdominal fat. However, our study also reveals that this association between equations used to estimate body composition and prediabetes was weak. Furthermore, our research was not able to find any anthropometric index that deserves to replace BMI in clinical practice.

Prediabetes is a metabolic condition with serious health consequences that needs to be screened [7]. As blood-based testing methods are expensive and time-consuming, non-invasive methods have been proposed, with particular interest in estimating body composition [10,12,33]. In fact, prediabetes and excess body weight are closely related, and the accumulation of adipose tissue in various depots has been linked to glucose abnormalities [34]. The National Health and Nutrition Examination Survey (1999–2004), after assessing body composition by DXA in 3888 participants, described a significant increase of trunk fat mass and in trunk/appendicular fat mass ratio with raised glucose intolerance (type 2 diabetes > impaired fasting glucose > normal glucose tolerance) [35]. Similarly, body magnetic resonance imaging showed a direct relationship between visceral adipose tissue and glucose tolerance status in 385 middle-aged subjects (53 with diabetes, 95 with prediabetes, and 237 controls) with no history of cardiovascular disease [36].

When three of the most popular anthropometric indices have been used, including BMI, WC and WHR, instead of standard gold techniques in general practice, similar results have been achieved [10,12,14,33]. The Spanish study PREDAPS recruited 1184 individuals with prediabetes (altered fasting plasma glucose and/or glycated hemoglobin) and 838 control subjects from primary care system [14]. After adjusting for confounding factors (age, family history of diabetes, smoking, alcohol, lipid lowering, blood hypertension, and dyslipidemia), abdominal obesity based on the WHR criterion in women and the WC in men showed the strongest association with prediabetes [odds ratio (OR): 2.48 (95% CI: 1.85–3.33) and 2.33 (1.75–3.08), respectively] [14]. In another cross-sectional study conducted in Venezuela with 2230 participants (19.5% with impaired fasting glucose), WHR was the most important predictor in both women [area under the ROC: 0.631 (95% CI: 0.588–0.673) and men (0.637 (0.596–0.678], with BMI and WC showing comparable predictive power [33]. The relationship between anthropometric indices and prediabetes also exists in non-obese populations. In this way, in the cross sectional *China National Diabetes and Metabolic Disorders Study*, with 10,098 men and 17,454 women with a BMI < 25 kg/m^2^, for each increase in the standard deviation of BMI (2.1 kg/m^2^) and WC (8.3 cm), fasting glucose levels increased by 0.128 and 0.170 mmol/L in men, and by 0.112 and 0.167 mmol/L in women, respectively [11]. However, neither BMI nor WC were significant risk factors for the prediction of prediabetes after 10 years of follow-up in a population-based cohort study with 1765 participants [13].

In our study, total adiposity was examined using CUN-BAE and Deurenberg’s equations together with BMI. Although there was no prior information on these two indices and the presence of prediabetes, our results showed a similar predictive power between the three indices, in both men and women.

Regarding the estimation of abdominal obesity and the prevalence of prediabetes, excluding WC and WHR, absent or scarce information is available [10,11,12,13,14,37,38]. To the best of our knowledge, there is no prior data concerning the Bonora equation and assessment of prediabetes. In the *2015 Health, Well-Being, and Aging Study*, with a total of 3307 Colombian individuals over the age of 60, there were significant differences in the BMI, body roundness index and conicity index between healthy patients and subjects with prediabetes, but with limited prediction capacity [39]. However, and likewise in our study, Ramírez-Vélez et al. failed to observe differences between groups regarding A body shape index [39]. In addition, in a large Chinese population including 15,078 participants, the body roundness index exhibited a highest area under the ROC for prediabetes than the BMI [40]. In the same study, A body shape index was associated with the lowest area under the ROCs for prediabetes in both sexes [40]. Thus, and in accordance with our results, this index seems to be the least appropriate to estimate the presence of prediabetes in the general population.

The neck circumference requires some additional comment. It is a simple and reliable anthropometric measure that has been proposed as a metabolic risk marker [41]. In a population of 1206 overweight and obese Hispanics, aged 40 to 65 free of major cardiovascular disease and type 2 diabetes, neck circumference was significantly associated with body fat percentage and showed a higher association with prediabetes compared to WC [OR 2.30 (95% CI: 1.71–3.06) vs. 1.97 (95% CI: 1.48–2.66)] [42]. In the ILERVAS population, neck circumference exhibited the higher correlation with HbA1c among anthropometric indices, but the area under the ROC was also a fair one. Therefore, the potential implications for the daily clinical practice of total body and abdominal adiposity measurements need further evaluation.

Some limitations of our research should be noted. First, the lack of an accurate measure of body composition determined with a DXA or magnetic resonance imaging. In fact, among 1603 Korean adults, the visceral fat mass measured with DXA had a higher OR for prediabetes than BMI, WC and WHR [43]. Second, we have not assessed other obesity indices including the hip circumference, like abdominal volume index, body adiposity index, and waist to hip ratio. The predictive capacity of prediabetes has been previously evaluated with these indices, and it remains to be clarified whether its inclusion can improve our results [44,45]. Third, we used HbA1c as a diagnostic tool for prediabetes. We are not sure whether the inclusion of impaired fasting glucose assessment and impaired glucose tolerance 2 h after a 75 g oral glucose tolerance test would change our results. Fourth, our study population with prediabetes is characterized by having at least one cardiovascular risk factor. While this is an intrinsic feature of the design of the ILERVAS study, we must be cautious when generalizing our results to other subjects with prediabetes in the general population. Finally, we have not been able to create a causal association concerning anthropometric indices and prediabetes due to the cross-sectional design of our research.

In closing, the main finding of our work was the lack of differences between the indices related to total body or abdominal adiposity to better categorize the presence of prediabetes. Moreover, total area under ROC indicates that none of them were useful in making clinical decisions in the ILERVAS population owing to low statistical power. Consequently, no isolated obesity index appears to be the perfect biomarker to use in clinical practice to detect subjects at risk for prediabetes. Even so, we recommend narrowing prediabetes screening in subjects with some cardiovascular risk factor and moderate overweighed (in our cohort, a BMI ≥ 28.2 kg/m^2^ in males ≥ 28.6 kg/m^2^ in females). Additional studies are needed, using the combination of new indices and the addition of other capital factors related with glucose abnormalities such as patient’s physical activity or patient phenotype to improve the explanatory power of anthropometric indices for glucose abnormalities.

## Figures and Tables

**Table 1:** Main clinical and metabolic data in the study population according to the presence of prediabetes.

	Control Group (*n* = 5457)	Prediabetes (*n* = 2731)	*p*-Value
Women, *n* (%)	2616 (47.9)	1551 (56.8)	<0.001
Age (years)	56 (52–62)	59 (54–64)	<0.001
Caucasian, *n* (%)	5440 (99.7)	2712 (99.3)	0.013
HbA1c (%)	5.4 (5.2–5.5)	5.8 (5.7–6.0)	<0.001
Obesity, *n* (%)	1580 (29.0)	1094 (40.1)	<0.001
Blood hypertension, *n* (%)	2015 (36.9)	1290 (47.2)	<0.001
Systolic BP (mm Hg)	129 (119–141)	132 (121–143)	<0.001
Diastolic BP (mm Hg)	81 (75–88)	82 (75–88)	0.184
Antihypertensive drugs, *n* (%)	1569 (28.8)	1103 (40.4)	<0.001
Dyslipidemia, *n* (%)	2798 (51.3)	1622 (59.4)	<0.001
Total cholesterol (mg/dL)	202 (179–229)	206 (183–232)	<0.001
Lipid-lowering agents, *n* (%)	831 (15.2)	646 (23.7)	<0.001
Antithrombotic drugs, *n* (%)	141 (2.6)	106 (3.9)	0.001

Data are expressed as a median [interquartile range] or *n* (percentage). HbA1c: glycated hemoglobin; BP: blood pressure. Obesity was defined as a body mass index ≥30 kg/m^2^. Antihypertensive drugs include angiotensin-converting enzyme (ACE) inhibitors, diuretics, angiotensin-II receptor antagonists (ARA II), beta-blockers, calcium antagonists, and other antihypertensives. Lipid-lowering treatments involve statins, fibrates, ezetimibe, and omega-3 fatty acids. Antithrombotic drugs include anticoagulants and antiplatelets.

**Table 2:** Results of the anthropometric indices in male and female participants according to the presence of prediabetes.

Male Subjects	Control Group (*n* = 2841)	Prediabetes (*n* = 1180)	*p*-Value
Total adiposity			
BMI (Kg/m^2^)	28.1 (25.6–31.0)	29.8 (27.4–33.0)	<0.001
CUN-BAE (%)	29.5 (26.2–33.1)	31.7 (28.7–35.3)	<0.001
Deurenberg (%)	30.1 (27.0–33.9)	32.6 (29.3–36.4)	<0.001
Visceral adipose tissue			
WC (cm)	100 (94–107)	104 (98–112)	<0.001
Conicity index	1.33 (1.29–1.37)	1.34 (1.31–1.39)	<0.001
WHR	0.59 (0.55–0.63)	0.60 (0.58–0.66)	<0.001
Bonora (cm^2^)	183 (145–228)	209 (171–260)	<0.001
A body shape index	0.08 (0.08–0.09)	0.08 (0.08–0.09)	0.242
Body roundness index	5.04 (4.27–6.00)	5.63 (4.82–6.72)	<0.001
Neck circumference (cm)	40.5 (39.0–42.0)	41.5 (39.5–43.5)	<0.001
**Female Subjects**	**(*n* = 2616)**	**(*n* = 1551)**	
Total adiposity			
BMI (Kg/m^2^)	27.3 (24.3–30.9)	29.5 (26.2–33.3)	<0.001
CUN-BAE (%)	41.0 (37.1–44.9)	43.6 (39.8–47.3)	<0.001
Deurenberg (%)	41.2 (37.0–45.7)	44.1 (40.1–48.7)	<0.001
Visceral adipose tissue			
WC (cm)	97 (90–105)	102 (94–110)	<0.001
Conicity index	1.36 (1.30–1.41)	1.37 (1.32–1.42)	<0.001
WHR	0.62 (0.57–0.67)	0.65 (0.60–0.70)	<0.001
Bonora (cm^2^)	177.7 (144.8–213.5)	199.4 (165.7–234.2)	<0.001
A body shape index	0.09 (0.08–0.09)	0.09 (0.08–0.09)	0.179
Body roundness index	5.80 (4.65–7.03)	6.47 (5.37–7.87)	<0.001
Neck circumference (cm)	34.5 (33.0–36.0)	35.5 (34.0–37.5)	<0.001

Data are expressed as a median [interquartile range]. BMI: body mass index; WC: waist circumference; WHR: waist to height ratio; CUN-BAE: Clínica Universidad de Navarra—Body Adiposity Estimator.

**Table 3:** Bivariate correlations of glycated hemoglobin with anthropometric indices in the participants with prediabetes according to sex distribution.

	Male Subjects		Female Subjects	
	r	*p*	r	*p*
BMI (Kg/m^2^)	0.152	<0.001	0.156	<0.001
CUN-BAE (%)	0.161	<0.001	0.161	<0.001
Deurenberg (%)	0.171	<0.001	0.164	<0.001
Waist circumference (cm)	0.138	<0.001	0.147	<0.001
Conicity index	0.095	0.001	0.083	0.001
WHR	0.149	<0.001	0.156	<0.001
Bonora (cm^2^)	0.136	<0.001	0.158	<0.001
A body shape index	0.018	0.542	0.007	0.777
Body roundness index	0.149	<0.001	0.156	<0.001
Neck circumference (cm)	0.178	<0.001	0.182	<0.001

BMI: body mass index; CUN-BAE: Clinica Universidad de Navarra-Body Adiposity Estimator; WHR: waist to height ratio.

**Table 4:** Receiver Operating Characteristic curves and appropriate cutoff of anthropometric indices for predicting prediabetes according to sex distribution.

Male Subjects	Cutoff	Sensitivity	Specificity	AUROC	95% CI	*p*
BMI (Kg/m^2^)	28.2	0.68	0.51	0.62	0.60 to 0.64	<0.001
CUN-BAE (%)	29.9	0.66	0.53	0.63	0.61 to 0.65	<0.001
Deurenberg (%)	31.5	0.59	0.60	0.63	0.61 to 0.65	<0.001
WC (cm)	100	0.65	0.53	0.62	0.60 to 0.64	<0.001
Conicity index	1.33	0.62	0.49	0.58	0.56 to 0.60	<0.001
WHR	0.59	0.65	0.54	0.63	0.61 to 0.65	<0.001
Bonora (cm^2^)	186.5	0.65	0.53	0.62	0.60 to 0.64	<0.001
Body roundness index	5.29	0.62	0.57	0.63	0.61 to 0.65	<0.001
A body shape index	0.08	0.50	0.54	0.51	0.49 to 0.53	0.228
Neck circumference (cm)	40.8	0.63	0.55	0.61	0.59 to 0.63	<0.001
**Female Subjects**						
BMI (Kg/m^2^)	28.6	0.58	0.60	0.62	0.60 to 0.64	<0.001
CUN-BAE (%)	43.5	0.51	0.67	0.63	0.61 to 0.64	<0.001
Deurenberg (%)	40.8	0.71	0.47	0.63	0.61 to 0.65	<0.001
WC (cm)	101.5	0.51	0.64	0.60	0.59 to 0.62	<0.001
Conicity index	1.35	0.59	0.48	0.55	0.53 to 0.57	<0.001
WHR	0.62	0.62	0.54	0.62	0.60 to 0.63	<0.001
Bonora (cm^2^)	179.0	0.67	0.51	0.62	0.60 to 0.63	<0.001
Body roundness index	6.10	0.59	0.57	0.62	0.60 to 0.63	<0.001
A body shape index	0.08	0.58	0.39	0.49	0.47 to 0.51	0.179
Neck circumference (cm)	35.3	0.55	0.63	0.62	0.60 to 0.64	<0.001

AUROC: area under the receiver operating characteristic; BMI: body mass index; WC: waist circumference; WHR: waist to height ratio; CUN-BAE: Clínica Universidad de Navarra-Body Adiposity Estimator.

**Table 5:** Prevalence of prediabetes above and below the cutoff proposed for each anthropometric index.

Male Subjects	Cutoff Point	Prevalence of Prediabetes Below the Cutoff	Prevalence of Prediabetes Above the Cutoff	Odds Ratio (95% CI)	*p*-Value
BMI (Kg/m^2^)	28.2	20.4	36.2	2.2 (1.9 to 2.5)	<0.001
CUN-BAE (%)	29.9	20.9	37.1	2.2 (1.9 to 2.6)	<0.001
Deurenberg (%)	31.5	22.8	38.2	2.2 (1.9 to 2.5)	<0.001
WC (cm)	100	20.8	35.4	2.1 (1.8 to 2.4)	<0.001
Conicity index	1.33	24.3	33.6	1.6 (1.4 to 1.8)	<0.001
WHR	0.59	21.0	36.8	2.2 (1.9 to 2.5)	<0.001
Bonora (cm^2^)	186.5	21.7	36.2	2.0 (1.8 to 2.4)	<0.001
Body roundness index	5.29	21.5	37.8	2.2 (1.9 to 2.6)	<0.001
A body shape index	0.08	27.3	29.8	1.1 (0.9 to 1.4)	0.204
Neck circumference (cm)	40.8	22.0	36.6	2.0 (1.8 to 2.4)	<0.001
**Female Subjects**					
BMI (Kg/m^2^)	28.6	29.3	44.8	2.0 (1.7 to 2.2)	<0.001
CUN-BAE (%)	43.5	30.4	47.8	2.1 (1.8 to 2.4)	<0.001
Deurenberg (%)	40.8	26.8	44.4	2.2 (1.9 to 2.5)	<0.001
WC (cm)	101.5	31.3	45.5	1.8 (1.6 to 2.1)	<0.001
Conicity index	1.35	33.2	40.0	1.3 (1.2 to 1.5)	<0.001
WHR	0.62	29.2	43.6	1.8 (1.6 to 2.1)	<0.001
Bonora (cm^2^)	179.0	28.2	44.4	2.0 (1.8 to 2.3)	<0.001
Body roundness index	6.10	29.9	45.0	1.9 (1.7 to 2.2)	<0.001
A body shape index	0.08	37.8	37.1	0.9 (0.8 to 1.2)	0.749
Neck circumference (cm)	35.3	30.1	46.3	2.2 (1.7 to 2.3)	<0.001

BMI: body mass index; WC: waist circumference; CUN-BAE: Clínica Universidad de Navarra-Body Adiposity Estimator.

## Data Availability

The data that support the findings of this study are available from the ILERVAS project and the corresponding author (Albert Lecube) upon reasonable request.

## Supplementary Materials

The following are available online at https://www.mdpi.com/2072-6643/13/3/1002/s1, Table S1. Results of the anthropometric indices in the entire population according to the presence of prediabetes. Table S2. Receiver Operating Characteristic curves and appropriate cutoff of anthropometric indices for predicting prediabetes in the entire population.

## References

[1] Cho N., Shaw J., Karuranga S., Huang Y., Fernandes J.D.R., Ohlrogge A., Malanda B. (2018). IDF Diabetes Atlas: Global estimates of diabetes prevalence for 2017 and projections for 2045. Diabetes Res. Clin. Pract..

[2] DeFronzo R.A., Abdul-Ghani M.A. (2011). Preservation of β-Cell Function: The Key to Diabetes Prevention. J. Clin. Endocrinol. Metab..

[3] Lee C.C., Perkins B.A., Kayaniyil S., Harris S.B., Retnakaran R., Gerstein H.C., Zinman B., Hanley A.J. (2015). Peripheral Neuropathy and Nerve Dysfunction in Individuals at High Risk for Type 2 Diabetes: The PROMISE Cohort. Diabetes Care.

[4] Huang Y., Cai X., Mai W., Li M., Hu Y. (2016). Association between prediabetes and risk of cardiovascular disease and all cause mortality: Systematic review and meta-analysis. BMJ.

[5] Sánchez E., Collaborators T.I.P., Betriu À., López-Cano C., Hernández M., Fernández E., Purroy F., Bermúdez-López M., Farràs-Sallés C., Barril S. (2019). Characteristics of atheromatosis in the prediabetes stage: A cross-sectional investigation of the ILERVAS project. Cardiovasc Diabetol..

[6] Sánchez E., Project I., Gutiérrez-Carrasquilla L., Barbé F., Betriu À., López-Cano C., Gaeta A.M., Purroy F., Pamplona R., Ortega M. (2019). Lung function measurements in the prediabetes stage: Data from the ILERVAS Project. Acta Diabetol..

[7] American Diabetes Association 2 (2021). Classification and Diagnosis of Diabetes: Standards of Medical Care in Diabetes—2021. Diabetes Care.

[8] Machann J., Stefan N., Wagner R., Fritsche A., Bell J.D., Whitcher B., Häring H., Birkenfeld A.L., Nikolaou K., Schick F. (2020). Normalized Indices Derived from Visceral Adipose Mass Assessed by Magnetic Resonance Imaging and Their Correlation with Markers for Insulin Resistance and Prediabetes. Nutrients.

[9] Mahat R.K., Singh N., Arora M., Rathore V. (2019). Health risks and interventions in prediabetes: A review. Diabetes Metab. Syndr. Clin. Res. Rev..

[10] Zhao X., Zhu X., Zhang H., Zhao W., Li J., Shu Y., Li S., Yang M., Cai L., Zhou J. (2012). Prevalence of diabetes and predictions of its risks using anthropometric measures in southwest rural areas of China. BMC Public Health.

[11] Li S., Xiao J., Ji L., Weng J., Jia W., Lu J., Zhou Z., Guo X., Liu J., Shan Z. (2014). BMI and waist circumference are associated with impaired glucose metabolism and type 2 diabetes in normal weight Chinese adults. J. Diabetes its Complicat..

[12] Haghighatdoost F., Amini M., Feizi A., Iraj B. (2017). Are body mass index and waist circumference significant predictors of diabetes and prediabetes risk: Results from a population based cohort study. World J. Diabetes.

[13] Zhang F., Wan Q., Cao H., Tang L., Li D., Lü Q., Yan Z., Li J., Yang Q., Zhang Y. (2018). Identical anthropometric characteristics of impaired fasting glucose combined with impaired glucose tolerance and newly diagnosed type 2 diabetes: Anthropometric indicators to predict hyperglycaemia in a community-based prospective cohort study in southwest China. BMJ Open..

[14] Sangrós F.J., Torrecilla J., Giráldez-García C., Carrillo L., Mancera J., Mur T., Franch J., Díez J., Goday A., Serrano R. (2018). Association of General and Abdominal Obesity With Hypertension, Dyslipidemia and Prediabetes in the PREDAPS Study. Rev. Española de Cardiol. (Engl. Ed.).

[15] Blundell J.E., Dulloo A.G., Salvador J., Frühbeck G. (2014). Beyond BMI-Phenotyping the Obesities. Obes. Facts.

[16] Ortega F.B., Sui X., Lavie C.J., Blair S.N. (2016). Body Mass Index, the Most Widely Used but Also Widely Criticized Index: Would a Criteri-on Standard Measure of Total Body Fat Be a Better Predictor of Cardiovascular Disease Mortality?. Mayo Clin. Proc..

[17] Kelly T.L., Wilson K.E., Heymsfield S.B. (2009). Dual Energy X-Ray Absorptiometry Body Composition Reference Values from NHANES. PLoS ONE.

[18] Gomez-Ambrosi J., Silva C., Catalan V., Rodriguez A., Galofre J., Escalada J., Valenti V., Rotellar F., Romero S., Ramirez B. (2011). Clinical Usefulness of a New Equation for Estimating Body Fat. Diabetes Care.

[19] Deurenberg P., Weststrate J.A., Seidell J.C. (1991). Body mass index as a measure of body fatness: Age- and sex-specific prediction formulas. Br. J. Nutr..

[20] Valdez R. (1991). A simple model-based index of abdominal adiposity. J. Clin. Epidemiol..

[21] Ashwell M., Gunn P., Gibson S. (2011). Waist-to-height ratio is a better screening tool than waist circumference and BMI for adult cardiometabolic risk factors: Systematic review and meta-analysis. Obes. Rev..

[22] Bonora E., Micciolo R., Ghiatas A.A., Lancaster J.L., Alyassin A., Muggeo M., DeFronzo R.A. (1995). Is it possible to derive a reliable estimate of human visceral and subcutaneous abdominal adipose tissue from simple anthropometric measurements?. Metabolism.

[23] Krakauer N.Y., Krakauer J.C. (2012). A New Body Shape Index Predicts Mortality Hazard Independently of Body Mass Index. PLoS ONE.

[24] Thomas D.M., Bredlau C., Bosy-Westphal A., Mueller M., Shen W., Gallagher D., Maeda Y., McDougall A., Peterson C.M., Ravussin E. (2013). Relationships between body roundness with body fat and visceral adipose tissue emerging from a new geometrical model. Obesity.

[25] Després J.P., Couillard C., Gagnon J., Bergeron J., Leon A.S., Rao D.C., Skinner J.S., Wilmore J.H., Bouchard C. (2000). Race, visceral adipose tissue, plasma lipids, and lipoprotein lipase activity in men and women: The Health, Risk Factors, Exercise Training, and Genetics (HERITAGE) family study. Arterioscler. Thromb. Vasc. Biol..

[26] Betriu À., Farràs C., Abajo M., Martinez-Alonso M., Arroyo D., Barbé F., Buti M., Lecube A., Portero M., Purroy F. (2016). Randomised intervention study to assess the prevalence of subclinical vascular disease and hidden kidney disease and its impact on morbidity and mortality: The ILERVAS project. Nefrología (Engl. Ed.).

[27] Bermúdez-López M., Martínez-Alonso M., Castro-Boqué E., Betriu À., Cambray S., Farràs C., Barbé F., Pamplona R., Lecube A., Mauricio D. (2020). Subclinical atheromatosis localization and burden in a low-to-moderate cardiovascular risk population: The ILERVAS study. Rev. Española de Cardiol. (Engl. Ed.).

[28] Lenters-Westra E., Slingerland R.J. (2014). Three of 7 Hemoglobin A1c Point-of-Care Instruments Do Not Meet Generally Accepted Analytical Performance Criteria. Clin. Chem..

[29] World Health Organization (2000). Obesity: Preventing and managing the global epidemic. Report of a WHO consultation. World Health Organ. Tech. Rep. Ser..

[30] Ma W.-Y., Yang C.-Y., Shih S.-R., Hsieh H.-J., Hung C.S., Chiu F.-C., Lin M.-S., Liu P.-H., Hua C.-H., Hsein Y.-C. (2013). Measurement of Waist Circumference: Midabdominal or iliac crest?. Diabetes Care.

[31] Ben-Noun L.L., Laor A. (2006). Relationship between changes in neck circumference and cardiovascular risk factors. Exp. Clin. Cardiol..

[32] Craig C.L., Marshall A.L., Sjöström M., Bauman A.E., Booth M.L., Ainsworth B.E., Pratt M., Ekelund U., Yngve A., Sallis J.F. (2003). International physical activity questionnaire: 12-country reliability and validity. Med. Sci. Sports Exerc..

[33] Bermúdez V., Salazar J., Rojas J., Calvo M., Rojas M., Chávez-Castillo M., Añez R., Cabrera M. (2016). Diabetes and Impaired Fasting Glucose Prediction Using Anthropometric Indices in Adults from Maracaibo City, Venezuela. J. Community Health.

[34] Hill J.O., Galloway J.M., Goley A., Marrero D.G., Minners R., Montgomery B., Peterson G.E., Ratner R.E., Sanchez E., Aroda V.R. (2013). Scientific Statement: Socioecological Determinants of Prediabetes and Type 2 Diabetes. Diabetes Care.

[35] Julian V., Blondel R., Pereira B., Thivel D., Boirie Y., Duclos M. (2017). Body Composition Is Altered in Pre-Diabetic Patients with Impaired Fasting Glucose Tolerance: Results from the NHANES Survey. J. Clin. Med. Res..

[36] Heber S.D., Hetterich H., Lorbeer R., Bayerl C., Machann J., Auweter S., Storz C., Schlett C.L., Nikolaou K., Reiser M. (2017). Pancreatic fat content by magnetic resonance imaging in subjects with prediabetes, diabetes, and controls from a general population without cardiovascular disease. PLoS ONE.

[37] Carba D.B., Bas I.N., Gultiano S.A., Lee N.R., Adair L.S. (2012). Waist circumference and the risk of hypertension and prediabetes among Filipino women. Eur. J. Nutr..

[38] Borné Y., Nilsson P.M., Melander O., Hedblad B., Engström G. (2015). Multiple anthropometric measures in relation to incidence of diabetes: A Swedish population-based cohort study. Eur. J. Public Health.

[39] Ramírez-Vélez R., Pérez-Sousa M.Á., González-Ruíz K., Cano-Gutierrez C.A., Schmidt-RioValle J., Correa-Rodríguez M., Izquierdo M., Romero-García J.A., Campos-Rodríguez A.Y., Triana-Reina H.R. (2019). Obesity- and Lipid-Related Parameters in the Identification of Older Adults with a High Risk of Prediabetes According to the American Diabetes Association: An Analysis of the 2015 Health, Well-Being, and Aging Study. Nutrients.

[40] Zhao Q., Zhang K., Li Y., Zhen Q., Shi J., Yu Y., Tao Y., Cheng Y., Liu Y. (2018). Capacity of a body shape index and body roundness index to identify diabetes mellitus in Han Chinese people in Northeast China: A cross-sectional study. Diabet. Med..

[41] Volaco A., Martins C.M., Soares J.Q., Cavalcanti A.M., Moyses S.T., Baena C.P., Precoma D.B. (2018). Neck Circumference and its Correlation to Other Anthropometric Parameters and Finnish Diabetes Risk Score (FINDRISC). Curr. Diabetes Rev..

[42] Joshipura K., Muñoz-Torres F., Vergara J., Palacios C., Pérez C.M. (2016). Neck Circumference May Be a Better Alternative to Standard Anthropometric Measures. J. Diabetes Res..

[43] Jung S.H., Ha K.H., Kim D.J. (2016). Visceral Fat Mass Has Stronger Associations with Diabetes and Prediabetes than Other Anthropometric Obesity Indicators among Korean Adults. Yonsei Med. J..

[44] Bala M., Meenakshi Aggarwal S. (2019). Correlation of Body Mass Index and Waist/Hip Ratio with Glycated Hemoglobin in Prediabetes. EJIFCC.

[45] Noudeh Y.J., Hadaegh F., Vatankhah N., Momenan A.A., Saadat N., Khalili D., Azizi F. (2013). Wrist Circumference as a Novel Predictor of Diabetes and Prediabetes: Results of Cross-Sectional and 8.8-Year Follow-up Studies. J. Clin. Endocrinol. Metab..

