# Supplementary material for: Clinical Usefulness of Anthropometric Indices to Predict the Presence of Prediabetes. Data from the ILERVAS Cohort
Source: Nutrients. 2021 Mar 19;13(3):1002. doi: 10.3390/nu13031002 (PMC8003825; doi:10.3390/nu13031002)
Supplement: Supplementary file 1 [file nutrients-13-01002-s001.pdf]

Supplementary Table 1. Results of the anthropometric indices in the entire population according to the presence of prediabetes.

	**Control group (n=5,457)**	**Prediabetes (n=2,731)**	**p-value**
Total adiposity			
BMI (Kg/m^2)	27.8 [24.9-31.0]	29.7 [26.7-33.2]	<0.001
CUN-BAE (%)	34.7 [29.1-41.1]	39.0 [32.4-44.7]	<0.001
Deurenberg (%)	35.2 [29.7-41.6]	39.5 [33.1-45.6]	<0.001
Visceral adipose tissue			
WC (cm)	99 [92-106]	103 [96-111]	<0.001
Conicity index	1.34 [1.30-1.39]	1.36 [1.31-1.41]	<0.001
WHR	0.60 [0.56-0.65]	0.63 [0.59-0.69]	<0.001
Bonora (cm^2)	183 [145-221]	203 [169-245]	<0.001
A body shape index	0.08 [0.08-0.09]	0.08 [0.08-0.09]	0.061
Body roundness index	5.37 [4.41-6.51]	6.07 [5.11-7.41]	<0.001
Neck circumference (cm)	38.0 [34.5-40.5]	38.0 [35.0-41.0]	<0.001

Data are expressed as a median [interquartile range]. BMI: body mass index; WC: waist circumference; WHR: waist to height ratio; CUN-BAE: Clínica Universidad de Navarra - Body Adiposity Estimator.

Supplementary Table 2. Receiver Operating Characteristic curves and appropriate cutoff of anthropometric indices for predicting prediabetes in the entire population.

	Cutoff	Sensitivity	Specificity	AUROC	95% CI	p-value
**BMI (Kg/m^2)**	28.7	0.59	0.57	0.61	0.59 to 0.62	<0.001
**CUN-BAE (%)**	34.7	0.67	0.49	0.61	0.60 to 0.63	<0.001
**Deurenberg (%)**	31.5	0.57	0.42	0.63	0.61 to 0.65	<0.001
**WC (cm)**	102	0.55	0.60	0.60	0.59 to 0.61	<0.001
**Conicity index**	1.33	0.68	0.42	0.57	0.56 to 0.58	<0.001
**WHR**	0.59	0.72	0.47	0.63	0.62 to 0.64	<0.001
**Bonora (cm^2)**	186	0.63	0.45	0.61	0.60 to 0.63	<0.001
**Body roundness index**	5.28	0.71	0.48	0.63	0.62 to 0.64	<0.001
**A body shape index**	0.08	0.43	0.59	0.51	0.49 to 0.52	0.147
**Neck circumference (cm)**	34.8	0.79	0.26	0.54	0.52 to 0.55	<0.001

AUROC: area under the receiver operating characteristic; BMI: body mass index; WC: waist circumference; WHR: waist to height ratio; CUN-BAE: Clínica Universidad de Navarra-Body Adiposity Estimator.
